# Neuroprotective effects of *Coriandrum sativum *and its constituent, linalool: A review

**DOI:** 10.22038/AJP.2021.55681.2786

**Published:** 2021

**Authors:** Mahmoud Hosseini, Mohammad Hossein Boskabady, Mohammad Reza Khazdair

**Affiliations:** 1 *Division of Neurocognitive Sciences, Psychiatry and Behavioral Sciences Research Center, Mashhad University of Medical Sciences, Mashhad, Iran*; 2 *Applied Biomedical* * Research Center, Mashhad University of Medical Sciences, Mashhad,* *Iran*; 3 *Cardiovascular Diseases Research Center, Birjand University of Medical Sciences, Birjand, Iran*

**Keywords:** Coriandrum sativum L., Neuro-pharmacological effects, Memory impairment, Linalool

## Abstract

**Objective:**

Coriander (*Coriandrum sativum* L.) is an annual herb belonging to the Apiaceae (Umbellifera) family that is used as food additives traditionally. This plant is called “*Geshniz*” in Persian and is native to Mediterranean regions but it is currently cultivated in several countries. All parts of coriander are edible and have been traditionally used to treat different disorders, including digestive problems, flatulence, diarrhea, colic and other gastrointestinal diseases.

**Materials and Methods:**

The databases PubMed, Web of Science, Google Scholar and Scopus were considered. The search terms were “*Coriandrum sativum*” or “linalool” and “anti-anxiety”, “sedative”, “antioxidant effect”, “anticonvulsant” and “neuroprotective effect”.

**Results:**

Antioxidant, diuretic, cholesterol lowering, anxiolytic, sedative-hypnotic and anticonvulsant activities were reported for the seeds and leaves of the plant. Furthermore, linalool as the main component of coriander has different neuropharmacological effects, including anti-anxiety, sedative, anticonvulsant and anti-Alzheimer’s disease activities.

**Conclusion:**

Various neuropharmacological effects of *C. sativum* and its component which have antioxidant and anti-inflammatory effects, have been summarized in the current review article.

## Introduction

**Figure 1:**
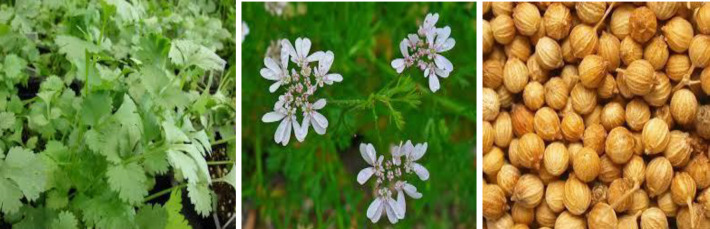
Leaf (left), flower (middle) and seeds (right) of coriander (Asgarpanah and Kazemivash, 2012)

Coriander (*Coriandrum sativum* L.) (C. sativum), is an annual herb originating from Mediterranean areas. The leaves are small branches and sub-branches, the flowers are white and the fruits are almost ovate globular, with multiple longitudinal ridges on the surface (Figure 1) (Yeung and Bowra, 2011 ▶). All segments of this plant are edible and have been traditionally used for treatment of different disorders (Sahib et al., 2013 ▶). The fresh leaves can be used for garnishing and are used in many foods like chutneys, salads and soups (Bhat et al., 2014 ▶). Green coriander contains 84% water, a low level of saturated fats, and cholesterol, but a high level of thiamine, zinc and dietary fibers (Kandlakunta et al., 2008 ▶). Coriander seeds are also considered a source of vitamins, lipids and minerals, such as potassium, calcium, phosphorus, magnesium, sodium and zinc (Bhat et al., 2014 ▶; Iwatani et al., 2003 ▶). The main coriander essential oil (EO) components are linoleic and linolenic acids (Sahib et al., 2013 ▶). Nutritional supplementation with coriander seeds is suggested to be able to decrease saturated but increase unsaturated fatty acids (Ertas et al., 2005 ▶).

Coriander is commonly used to attenuate digestive problems, flatulence, diarrhea and colic and other gastrointestinal diseases in Iranian traditional medicine (Zargari, 1991 ▶). Hepatoprotective properties of *C. sativum* due to the antioxidant potential of its phenolic compounds (Pandey et al., 2011 ▶) and cardiovascular protective effect of the plant were demonstrated (Dhanapakiam et al., 2007 ▶). The plant also showed antioxidant activities in different organs (Kansal et al., 2011 ▶; Wangensteen et al., 2004 ▶) including a preventive effect on the changes in the brain histology such as gliosis, lymphocytic infiltration and cellular edema (Vekaria et al., 2012 ▶). 

It has been reported that treatment with leaves of *C. sativum* (5, 10 and 15% w/w) improved memory scores and prevented the process of aging in mice dose–dependently, reversed the memory deficits induced by scopolamine and diazepam and significantly reduced brain cholinesterase activity and serum total cholesterol levels (Mani et al., 2011 ▶). The preventive effect of the hydro-alcoholic extract of *C. sativum* (50, 100 and 200 mg/kg) on neuronal damages induced by pentylenetetrazole (PTZ) in a rat model of seizure, was also demonstrated (Pourzaki et al., 2017 ▶). Ethanolic extract of *C. sativum* seeds on learning in second-generation mice was assessed but the plant extract did not improve learning in a short period of time after training; however, it improved learning in long term (Zargar-Nattaj et al., 2011 ▶).

There is currently an increasing interest to discover effective drugs based on folk medicinal and medicinal plant. It should be noted that many modern drugs have plants origin, and were developed through observation of therapeutic practices of native people (Balick and Cox, 1996 ▶; Gilani, 2005 ▶). Therefore, in the present article, neuropharmacological properties of *C. sativum* and its component including their sedative, anti-anxiety, anti-depressive, anticonvulsant and anti-seizures, memory improving, anti-Parkinson’s diseases properties and protective effects on neurotoxicity, were reviewed. 

**Chemical composition of** ***C. sativum*** **essential oil**

*C. sativum* is cultivated in different soil, climatic, seasonal and other ecological conditions and therefore, has a broad range of compositions. Different essential oil (EO) components and different chemical compounds have been extracted from different parts of *C. sativum* which seems to be dependent to the variety of the plant (Diederichsen and Hammer, 2003 ▶). EO of premature fruits mainly contains compounds such as geranyl acetate (46.27%) and linalool (10.96%), while in mature fruits linalool (87.54%), followed by cis-dihydrocarvone (2.36%), were detected as the considerable compounds (Msaada et al., 2007 ▶).

The coriander seeds EO from different geographical origins also showed variations in constituents. The major components in Europe type of the plant are linalool (58.0−80.3%), γ-terpinene (0.3−11.2%) and α-pinene (0.2−10.9%) (Orav et al., 2011 ▶), while, the main oil components of the plant from Brazil were linalool (77.48%), γ-terpinene (4.64%) and α-pinene (5.5%) (de Figueiredo et al., 2004 ▶). Additionally, the seeds oil of the plant grown in Iran contain linalool (40.9−79.9%), neryl acetate (2.3−14.2%) and γ-terpinene (0.1−13.6%) (Nejad Ebrahimi et al., 2010 ▶).

The oils extracted from the leaves of the plant have been shown to contain 44 compounds, most of them are aromatic acids, including 2-decenoic (30.82%) and E-11-tetradecenoic acids (13.4%). Capric acid (12.7%) and undecyl alcohol (6.4%). Other compounds including 2-undecenal (3.87%), dodecanoic acid (2.63%), cyclododecane (2.45%), decanal (1.35%) and decamethylene glycol (1.15%) were also reported to be present in the leaves of the plant (Nurzyńska-Wierdak, 2013 ▶). Monoterpenoids such as limonene and beta-myrcene are also reported as the constituents of coriander EO (Tashinen and Nykänen, 1975 ▶).

**Sedative, anti-anxiety and anti-depressive properties of the plant and its component**

***C. sativum***

*C. sativum* has been recommended for relieving insomnia in Iranian folk medicine (Mir, 1992 ▶; Zargari, 1991 ▶). Pre-sleep eating of a combination of chopped fresh leaves (30 g) or seeds of the plant with tea, as a single dose, has been suggested to relieve anxiety and insomnia (Mir, 1992 ▶). Similar uses have also been advised in other folk medicines (Duke, 2002 ▶). These effects were also confirmed using animal models. Intraperitoneal (i.p.) administration of aqueous extracts (200, 400 and 600 mg/kg) and hydroalcoholic extracts (400 and 600 mg/kg), as well as 600 mg/kg EO of coriander in male albino mice increased sleep duration to 160-220, 130-180 min and 210 min, respectively. These results showed that the extracts and EO of coriander exhibit sedative and hypnotic activities in pentobarbital-induced hypnotic model (Emamghoreishi and Heidari-Hamedani, 2015 ▶). In another study, the sleep-prolonging effects of the fractions of aerial parts of the plant including water (WF), ethyl acetate (EAF) and n-butanol (NBF) fractions were also confirmed in mice. The hydro-alcoholic extract (HAE), EAF and NBF significantly prolonged sleep duration but only the NBF decreased sleep latency significantly (Rakhshandeh et al., 2012 ▶). Using an animal model of anxiety, the effects of 10, 25, 50 and 100 mg/kg (i.p.) of aqueous extract of the seeds on motor activity and neuromuscular function have been evaluated. In elevated plus-maze, the seeds’ aqueous extract at 100 mg/kg (i.p.) showed an anxiolytic effect by increasing the time spent in and the entries to the open arms. Furthermore, administration of the extract (50, 100 and 500 mg/kg) was accompanied with motor activity impairments and neuromuscular dysfunction (Emamghoreishi et al., 2005 ▶). In another study, anti-anxiety effects of 100 and 200 mg/kg of hydroalcoholic extract of the seeds were comparable to the effects of 0.5 mg/kg diazepam, however, 50 mg/kg of the extract did not show an anti-anxiety activity (Mahendra and Bisht, 2011 ▶). The antianxiety activity of 50, 100 and 200 mg/kg (i.p.) of *C. sativum* leaves aqueous extract has also been evaluated in mice, and was compared with the effects of diazepam (5 mg/kg, i.p.) as a standard drug. Treatment by 200 mg/kg of leaves extract increased the time spent in and the number of entries into the open arms of elevated plus maze which was assumed to be due to anxiolytic effects of the extract (Pathan et al., 2011 ▶). Interestingly, 100 and 200 mg/kg of the fruit extract also had an anxiolytic effect which was similar to the effects of the leave extract. Locomotion activity and frequency of rearing in the groups treated by 200 mg/kg of the extract were also lower than that of the control group. Furthermore, 100 and 200 mg/kg of the plant extract significantly increased social interactions of treated animals (Mahendra and Bisht, 2011 ▶). In another study, 10 ml/kg (i.p.) of an extract obtained from the fresh plant attenuated immobility time and was more effective than the extract of dried plant. It was concluded that the extract of green but not dried plant have anti-depressive effect (Kishore and Siddiqui, 2003 ▶). 

Using tail suspension and forced swimming tests, aqueous extract and ﬁxed oil of the seeds showed anti-depressant activity at the end of 14 consecutive days of treatment. Treatment by aqueous extract 200 and 400 mg/kg, orally (p.o.) and also by 2 and 4 ml/kg (p.o.) of an extract prepared by diethyl ether showed a signiﬁcant anti-depressant like activity, which was similar to the effects of commonly used anti-depressant drugs ﬂuoxetine 20 mg/kg (p.o.) and imipramine 15 mg/kg (p.o.). Aqueous extract from the seeds also inhibited monoamine oxidase-B (MAO-B) activity, which was suggested as a mechanism of anti-depressant activity (Kharade et al., 2011 ▶). Essential oil from coriander seeds and its major component linalool has also been administered intracerebroventricularly (i.c.v) to examine their effects on locomotor activity in chicks. Similar to diazepam, coriander oil and linalool (0.86, 8.6 and 86 μg/chick, i.c.v. for both agent) significantly decreased escapes, defecations and crossing numbers in open field, while increased the sleeping posture (Gastón et al., 2016 ▶). 

The chemical composition of *C. sativum* volatile oil included linalool (69.358%), as the major component, γ-terpinene (7.729%), and α-pinene (6.509%). Inhalation of coriander oil (1 and 3%) significantly increased time spent in the open arms, open-arm entries and the number of crossing (exploratory locomotor activity) in a β-amyloid (1–42) rat model of Alzheimer's disease (Cioanca et al., 2014 ▶). *C. sativum* oil also reduced significantly immobility time, but increased swimming time and content of glutathione. Furthermore, amyloid deposits were scarce in rats treated with *C. sativum* oil, which were obvious and abundant in the hippocampus of rats treated with i.c.v. β-amyloid (1–42) (Cioanca et al., 2014 ▶).

The hydro-alcoholic extract of *C. sativum* (50, 100 and 200 mg/kg) reduced duration, frequency and amplitude of the burst discharges while prolonged the latency of the seizure attacks. The extract also prevented from production of dark neurons and apoptotic cells in different areas of the hippocampus (Pourzaki et al., 2017 ▶) (Table 1). 

***Linalool***

Inhalation of 1 and 3% of linalool increased the sleeping time and induced similar effects as 1 mg/kg diazepam. It also reduced body temperature but was not able to affect the bar descent latency in rota-rod performance test. Linalool 3% also signiﬁcantly impaired exploratory and motor performances in spontaneous locomotor activity evaluation (de Moura Linck et al., 2009 ▶).

Similar to administration of 1 mg/kg (i.p.) diazepam, inhalation of 3% linalool increased the time spent in the light compartment of light/dark test box and increased the latency in the ﬁrst crossing between the light and dark compartments, but had no effect on the crossing number (Linck et al., 2010 ▶). Both diazepam and linalool (1%) improved the social interaction behaviors. Furthermore, both linalool 3% and diazepam postponed the ﬁrst attack and significantly reduced the number and duration of the attacks in the aggressive behavior test (Linck et al., 2010 ▶). 

In elevated plus-maze test, inhalation of 0.65, 2.5 and 5.0% mass fraction (w/w) of linalool oxide and injection of 0.5 mg/kg of diazepam (i.p.) were able to increase the open arm entries and the time spent in open arms. Additionally, both linalool oxide and diazepam prolonged the time spent in the light compartment, as well as the crossing numbers between the light and dark compartments of light-dark box (Souto-Maior et al., 2011 ▶). Intraperitoneal administration of linalool (30 mg/kg) showed a significant antidepressant activity by increasing the latency time and self-cleaning time, while decreased immobility time in rats (dos Santos et al., 2018 ▶). Linalool (100 mg/kg, i.p.) significantly improved the cognitive performance in Morris water maze (MWM) test in an amyloid-beta (Aβ1-40)-induced model of cognitive deficits in mice. Treatment with linalool significantly decreased the prolonged time in dark chamber in mice. Treatment with linalool also reduced the pathological lesions induced by Aβ to normal range. In addition, linalool (100 mg/kg) significantly increased superoxide dismutase (SOD) and glutathione peroxidase (GPX) activities in the hippocampus and cortex compared to the induced animals, while decreased the MDA level in the cortex close to the sham group (Xu et al., 2017 ▶). Oral administration of linalool (25 mg/kg), every 48 hr for 3 months, improved learning and spatial memory and behavior in the elevated plus maze in a mouse model of Alzheimer's disease. Linalool (25 mg/kg) also significantly reduced extracellular β-amyloidosis, astrogliosis and microgliosis and reduced the levels of the pro-inflammatory markers including; p38 mitogen-activated protein kinases (p38 MAPK), cyclooxygenase-2 (COX-2) and interleukin (IL)-1β in hippocampi and amygdalae (Sabogal-Guáqueta et al., 2016 ▶) (Table 1). 

**Anticonvulsant and anti-seizure effects of** ***C. sativum*** **and linalool**

***C. sativum***

Anticonvulsant activity of aqueous and ethanolic extracts of coriander seeds was studied using electroshock and PTZ– induced seizure models. Administration of 0.5 g/kg (i.p.) of aqueous and 3.5 and 5 g/kg (i.p.) of ethenolic extracts shortened tonic seizures period and inhibited maximal electroshock-induced seizures. In addition, both of the extracts postponed the onset of clonic convulsions. The ethanolic extract effect was similar to that of 20 mg/kg of phenobarbital in the PTZ test (Hosseinzadeh and Madanifard, 2000 ▶). 

The effects of 5 mg/kg (i.p.) of the plant extract, as well as the effect of essential oil, on PTZ (85 mg/kg, i.p.)-induced convulsions were also assessed. Intraperitoneal administration of aqueous extracts, as well as essential oils, obtained from the seeds 30 min before PTZ injection, was effective to postpone myoclonic and clonic seizure onset in mice in a dose-dependent manner (Emam and Heydari, 2008 ▶). Similarly, 200, 400, 600 and 800 mg/kg of aqueous and ethanolic extracts, as well as essential oil, of the seeds were administered 30 min before PTZ injection (90 mg/kg, i.p.) to evaluate the anticonvulsant effects. The results showed that 600 and 800 mg/kg of all extracts increased myoclonic and clonic seizures latencies (Emamghoreishi and Heidari-Hamedani, 2010 ▶). 

The effect of leaves hydroalcoholic extract of coriander on oxidative damages in the brain tissues of PTZ- induced seizure was investigated (Karami et al., 2015 ▶). Administration of 100, 500 and 1000 mg/kg (i.p.) of the extract postponed the onset of the minimal clonic (MCS) and generalized tonic-clonic (GTCS) seizures. Furthermore, cortical and hippocampal tissues MDA levels in the animals pre-treated by the extract decreased significantly. 

**Table 1:** Sedative, anti-anxiety and anti-depressive effects of* C. sativum* and linalool

**Extract/Component**	**Type of study**	**Doses**	**Results**	**Ref.**	
Aqueous fresh leaves	Traditional medicine	-	Anxiety relieving and insomnia treating.	(Mir, 1992 ▶)	
Aqueous fresh leaves	Traditional medicine	-	Anxiety relieving and insomnia treating.		(Duke, 2002 ▶)
Aqueous seed extract	Elevated plus maze in mice	10, 25, 50, 100 mg/kg, i.p.	Increasing the time spent on elevated plus maze open arms motor activity and neuromuscular function impairments.	(Emamghoreishi et al., 2005 ▶)	
Aqueous seed extract	Pentobarbital-induced hypnosis in mice	100, 200, 400 and 600 mg/kg, i.p.	200, 400 and 600 mg/kg of the extract prolonged duration of sleeping time.	(Emamghoreishi and Heidari-Hamedani, 2015 ▶)	
Hydroalcoholic seed extract	Pentobarbital-induced hypnosis in mice	100, 200, 400 and 600 mg/kg, i.p.	400 and 600 mg/kg of the extract increased duration of sleeping time	(Emamghoreishi and Heidari-Hamedani, 2015 ▶)	
Essential oil of seed	Pentobarbital-induced hypnosis in mice	100, 200, 400 and 600 mg/kg, i.p.	Only 600 mg/kg of essential oil of seeds increased duration of sleeping time	(Emamghoreishi and Heidari-Hamedani, 2015 ▶)	
Aqueous leaves extract	Elevated plus maze in mice	50, 100 and 200 mg/kg, i.p.	200 mg/kg of the extract increased the time spent on and the number of entries into the open arms	(Pathan et al., 2011 ▶)	
Hydroalcoholic seed extract	Induced anxiety in mice	50, 100 and 200 mg/kg, i.p.	Similar to 0.5 mg/kg diazepam, 100 and 200 mg/kg of the extract attenuated anxiety like behaviors	(Mahendra and Bisht, 2011 ▶)	
An extract obtained from fresh and dried plant	Induced depression in mice	10 ml/kg, i.p.	The extract from fresh plant had an anti-depression effect	(Kishore and Siddiqui, 2003 ▶)	
Aqueous extracts and extract obtained by diethyl ether	Induced depression in mice	200 and 400 mg/kg, p.o. 2 and 4 ml/kg, p.o.	Showed anti-depressant like activity comparable to the effects of ﬂuoxetine and imipramine	(Kharade et al., 2011 ▶)	
Diethyl ether extract from the seeds	Induced depression in mice	6 and 8 ml/kg, p.o.	Showed better bioactivity than aqueous seeds extract	(Kharade et al. 2011 ▶)	
Coriander oil and linalool	Induced anxiety in chick	0.86, 8.6 and 86 μg/chick, i.c.v.	Distress calls, escapes, defecation and crossing number on open field test were decreased, while sleeping posture was increased	(Gastón et al., 2016 ▶)	
Inhalation coriander oil	Induced Alzheimer's disease in rat	1% and 3%	Increased time spent in the open arms, open-arm entries and the number of crossing in rat model of Alzheimer's disease. *C. sativum* oil also reduced immobility time, but increased swimming time and content of glutathione. Furthermore, reduced amyloid deposits in the hippocampus	(Cioanca et al., 2014 ▶).	
*C. sativum *hydro-alcoholic extract	Induced seizure in rat	50, 100 and 200 mg/kg	Reduced duration, frequency and amplitude of the burst discharges while prolonged the latency of the seizure attacks. The extract also prevented from production of dark neurons and apoptotic cells in different areas of the hippocampus	(Pourzaki et al., 2017 ▶)	
Inhaled linalool	Induced anxiety in mice	1% and 3%	Linalool 3% decreased exploring and motor activities. Linalool (1% and 3%) increased sleeping time and reduced body temperature.	(de Moura Linck et al., 2009 ▶)	
Inhaled linalool	Induced anxiety in mice	1% and 3%	Linalool (3%) increased exploring behavior comparable to the effects of diazepam (1.0 mg/kg, i.p.) Linalool (1%) increased social interactions	(Linck et al., 2010 ▶)	
Inhaled linalool oxide	Induced anxiety in mice	0.65%, 2.5% and 5.0% (w/w)	Increased open entries and open arm duration and also increased the time spent on light compartment.	(Souto-Maior et al., 2011 ▶)	
linalool	Induced depression in rat	30 mg/kg, p.o.	Showed antidepressant activity by increased latency time and self-cleaning time, while decreased immobility time.	(dos Santos et al., 2018 ▶)	
Linalool	Induced anxiety in mice	100 mg/kg, i.p.	Improved the cognitive performance and decreased the prolonged time in dark chamber of model mice. Linalool also reduced the pathological lesions induced by AB to normal range. It also increased SOD and GPX activities in the hippocampus and cortex compared to the induced animals, while decreased the MDA level in the cortex close to the sham group	(Xu et al., 2017 ▶)	
linalool	Induced Alzheimer's disease in mice	25 mg/kg, p.o.	Improved learning and spatial memory and significantly reduced extracellular β-amyloidosis, astrogliosis and microgliosis as well as reduced the levels of the pro-inflammatory markers including; p38 MAPK, COX2 and IL-1β in hippocampi and amygdalae.	(Sabogal-Guáqueta et al., 2016 ▶)	

Pretreatment with 500 mg/kg of the extract also significantly improved total thiol contents in the cortical tissues (Karami et al., 2015 ▶). Similarly, the effects of 25 and 100 mg/kg (i.p.) of WF, NBF and EAF fractions of the plant on the brain tissues oxidative damages due to seizures induced by PTZ, were investigated. The GTCS latency in WF and EAF treated rats (100 mg/kg, i.p) was longer than that of PTZ group. Interestingly, WF, NBF, and EAF attenuated hippocampal MDA levels. Additionally, both WF and NBF increased cortical and hippocampal thiol contents (Anaeigoudari et al., 2016 ▶), (Table 2).

***Linalool***

Anticonvulsant effects of linalool on experimental seizure models related to glutamate including N-methyl-D-aspartate (NMDA), quinolinic acid-induced convulsions, and the behavioral and neurochemical factors related to PTZ-kindling were investigated. Linalool at 0.3 and 1.0 mM was able to modulate glutamate activation expression in the rat cortical membrane cells (*in vitro*). Linalool (350 mg/kg, i.p.) and diazepam also significantly prolonged the delay time to the onset of NMDA (270 mg/kg, i.p.)-induced seizures. Administration of linalool (15, 30 and 45 mM, i.c.v.) significantly inhibit quinolinic acid (9.2 mM, i.c.v.)-induced seizure attack as dose-dependent manner. In addition, linalool (2.2 and 2.5 g/kg, p.o) significantly inhibited and delayed the behavioral expression in experimental seizure models (Elisabetsky et al., 1999 ▶). In mice cortical synaptosomes, linalool (1.0 or 3.0 mM) reduced potassium-stimulated glutamate release as well as glutamate uptake (90%) (Brum et al., 2001 ▶) (Table 2).

**Table 2:** Anticonvulsant and anti-seizure activity of *C. sativum *and linalool

**Extract/ Component**	**Type of study**	**Doses**	**Results**	**Ref.**
The seeds aqueous extract	Induced seizure in mice	0.5 g/kg, i.p.	Tonic seizures duration was decreased, clonic convulsion onsets were postponed.	(Hosseinzadeh and Madanifard, 2000 ▶)
The seeds ethanolic extract		3.5 and 5 g/kg, i.p.	Tonic seizures duration was decreased, clonic convulsion onsets were postponed.	(Hosseinzadeh and Madanifard, 2000 ▶)
Aqueous and ethanolic extracts and essential oil		200, 400, 600 and 800 mg/kg, i.p.	Myoclonic and clonic seizures onsets were postponed.	(Emamghoreishi and Heidari-Hamedani, 2010 ▶)
The seeds hydroalcoholic extract and essential oil		5 mg/kg, i.p.	Myoclonic and clonic seizures onsets were postponed.	(Emam and Heydari, 2008 ▶)
The leaves hydroalcoholic extract	PTZ- induced seizure in rat	100, 500 and 1000 mg/kg, i.p.	MCS and GTCS latencies were increased Cortical tissues total thiol contents were improved.	(Karami et al., 2015 ▶)
Aerial parts fractions		25 and 100 mg/kg, i.p.	GTCS latency was significantly improved Hippocampal MDA concentrations were decreased cortical and hippocampal tissues thiol contents were improved.	(Anaeigoudari et al., 2016 ▶)
Linalool	*In vitro*	0.3 mM or 1.0 mM	Modulated glutamate activation expression in the rat cortex membrane cells.	(da-Silva et al., 1990 ▶)
Linalool	Quinolinic acid induced seizure in rat	350 mg/kg, i.p.	Linalool delayed of NMDA (270mg/kg, i.p.) induced seizures onsets which was comparable to the effects of diazepam.	(da-Silva et al. 1990 ▶)
Linalool	Quinolinic acid induced seizure in mic	1.0 or 3.0 mM	Reduced potassium-stimulated glutamate release as well as glutamate uptake (90%)	(Brum et al., 2001 ▶)

**Memory improving effects of** ***C. sativum*** **and its component**

***C. sativum***

Effects of daily inhalation of volatile oil (1 and 3%) for 21 days on spatial memory performance were assessed in an Aβ1-40-induced Alzheimer's disease model in rats. Volatile oils (both 1 and 3%) improved and reversed deleterious effects of (Aβ1-40 i.c.v.) injection and increased percentage of spontaneous alternations and number of arm entries, reduced working and improved errors in radial arm-maze task. Coriander volatile oil also improved hippocampal tissues oxidative stress markers in Aβ1-40-treated rats by increasing GPX levels and reduction of SOD, lactate dehydrogenase (LDH) and MDA levels in a dose-dependent manner. The amyloid deposits were scarce in volatile oil-treated animals. Also, cleavages of DNA were absent in the coriander oil groups (Cioanca et al., 2013 ▶). Similarly, both 1 and 3% of volatile oil increased the time spent in and the entering number into the open arms in elevated plus-maze compared to the Aß group. The volatile oil also enhanced the swimming time and decreased the immobility time in Alzheimeric rats. The antioxidant parameters such as catalase (CAT) activity decreased and total content of reduced glutathione (GSH) were increased in the hippocampal tissues of the rats (Cioanca et al., 2014 ▶).

The protective and therapeutic effects of coriander seed aqueous extract in an aluminum chloride (AlCl_3_)-induced animal model of Alzheimer's disease and its effects on the cortical pyramidal cells were investigated in male albino rats. Treatment with aqueous seed coriander extract (0.5 mg/kg, p.o) for one month after stopping AlCl_3_ (300 mg/kg, p.o) treatment, restored deleterious effects on the pyramidal cells including, dilatation of blood capillaries and presence of many shrunken pyramidal cells (Enas, 2010 ▶).

Anti-amnestic and anti-stress properties of *C. sativum* extract on vanillylmandelic acid (VMA) and ascorbic acid urinary levels in scopolamine-induced amnesia model were evaluated in rats. Daily administration of 100, 200 and 300 mg/kg of the extract one-hour former to exposure of animals to stress, decreased the stress-induced urinary levels of VMA, while increased excretion of ascorbic acid. Moreover, *C. sativum* extract dose-dependently reversed acquisition, retention and recovery impairments induced by scopolamine (1 mg/kg, i.p.) which was accompanied with lipid peroxidation inhibition in both liver and brain tissues (Koppula and Choi, 2012 ▶).

Oral administration of young and aged rats by the leaves extract (5, 10 and 15 % w/w) for 45 days, dose-dependently improved memory. The leaves extract also reversed effectively the memory impairments induced by scopolamine (0.4 mg/kg, i.p.) or diazepam (1 mg/kg, i.p.) administration in plus-maze and Hebb-Williams maze models for testing memory (Mani and Parle, 2009 ▶; Mani et al., 2011 ▶). 

In learning and memory impairment due to seizures induced by pilocarpine (30 mg/kg, i.p.), administration of 200 mg/kg (i.p.) of the extract for 7 consecutive days, increased the latencies of seizure onset which was accompanied with a decreased level of latency to find the platform in Morris water maze (Elahdadi-Salmani et al., 2015 ▶), (Table 3).

***Linalool***

Beneficial properties of linalool on triple transgenic Alzheimer's disease model (3xTg-AD) in mice were studied. Both non transgenic and the 3xTg-AD mice received 25 mg/kg of linalool (p.o.) every 48 hr for 3 months, signiﬁcantly better retention performance and swam a longer distance in the platform location compared to vehicle-treated 3xTg-AD mice. Linalool treatment successfully increased the time spent in and the entries into the open arms compared to vehicle-treated mice. Linalool treatment also delayed deposits and abundance of Aß peptide and signiﬁcantly reduced the Aß (1–40), (1–42) protein and pair helical ﬁlaments (PHFs) levels in the hippocampal tissues. Additionally, immunohistochemistry study showed that glial fibrillary acidic protein (GFAP) decreased in the CA1 area of the hippocampus, the entorhinal cortex (EC) and amygdala in mice. In addition, linalool signiﬁcantly reduced extracellular of Aß concentration, astroglia and microglia as well as the pro-inﬂammatory marker levels, including mitogen-activated protein kinases p38, nitric oxide (NO) synthase 2, cyclooxygenase-2 and interleukin 1 beta in the AD treated mice (Sabogal-Guáqueta et al. 2016 ▶).

The effects of linalool (1%) inhalation on acquisition memory in the step-down inhibitory avoidance test showed the anxiolytic properties, increased social interaction and reduced aggressive behavior as well as improved memory in comparison with the control mice (Linck et al., 2010 ▶), (Table 3).

**Anti-Parkinson effects**

The effects of 100 and 200 mg/kg of the seeds ethanolic extract were evaluated on orofacial dyskinesia induced by tacrine. Administration of 2.5 mg/kg of tacrine (i.p), induced some of orofacial dyskinesia symptoms including tongue protrusions (TP), vacuous chewing movements (VCM) and orofacial bursts (OB) 1 hr after administration. Pre-treatment by 100 and 200 mg/kg (p.o.) of the seed extract for 15 days considerably diminished VCM, TP and OB induced by tacrine, and improved locomotor activity and cognitive functions. In addition, administration of the extract significantly improved SOD, CAT and GSH levels, while attenuated lipid peroxidation (Mohan et al., 2015 ▶), (Table 3).

**Table 3:** Memory improving and anti-Alzheimer’s disease properties of *C. sativum* and linalool

**Extract/ Component**	**Type of study**	**Doses**	**Results**	**Ref.**
Coriander volatile oil	Induced Alzheimer's disease in rats	1 and 3%	Improved and reversed the deleterious effects of amyloid ß.	(Cioanca et al., 2013 ▶)
Coriander volatile oil		1 and 3%	Enhanced the swimming time and attenuated the immobility times	(Cioanca et al., 2014 ▶)
Aqueous seed extract		0.5 mg/kg, p.o.	Restored deleterious effects on pyramidal cells.	(Enas, 2010 ▶)
Aqueous fruit extract		100, 200 and 300 mg/kg	Decreased VMA urinary levels in stress-induced animal model while, increased excretion of ascorbic acid.	(Koppula and Choi, 2012 ▶)
Leaves mixed in diet		5, 10 and 15% (w/w)	Improved memory scores in aged and young rats.	(Mani and Parle, 2009 ▶; Mani et al., 2011 ▶)
Ethanolic leaves extract		200 mg/kg; i.p.	Increased the latencies of seizure onsets which was accompanied with a decreased latency to find the platform.	(Elahdadi-Salmani et al., 2015 ▶)
Ethanolic seeds extract		100 and 200 mg/kg, p.o.	Decreased VCM, TP and OB behaviors induced by tacrine, and improved locomotor activity and cognitive functions.	(Mohan et al., 2015 ▶)
Linalool	Induced Alzheimer's disease in mice	25 mg/kg, p.o.	Showed a higher retention performance.	(Sabogal-Guáqueta et al., 2016 ▶)
Inhaled linalool		1%	The social interactions were increased.	(Linck et al., 2010 ▶)

**The effects of** ***C. sativum*** **and its components on neurotoxicity**

***C. sativum***

The beneficial effects of the seeds hydroalcoholic extract on neurodegeneration induced by lead exposure were evaluated. Daily treatment by 250 and 500 mg/kg of the extract for seven days which was started after 4 weeks of lead exposure, increased reactive oxygen species (ROS) production and lipid peroxidation and diminished total protein carbonyl content in the cerebellum, hippocampus, frontal cortex and brain stem (Velaga et al., 2014 ▶). Hydro-alcoholic extract, WF, EAF and NBF (0.1, 0.2, 0.4, and 0.8 mg/ml) had a protective activity on neurons (PC12 cells) in glucose/serum deprivation-induced cytotoxicity. The extract and the fractions showed no cytotoxicity in standard conditions while EAF or NBF (1.6 mg/ml) decreased cell survival. On the other hand, glucose/serum deprivation induced cytotoxicity was significantly reduced by WF at 0.4, 0.8 and 1.6 mg/ml (Ghorbani et al., 2011 ▶).

The leaves methanolic extract (200 mg/kg, p.o., per day) increased endogenous enzyme levels such as SOD, glutathione, catalase and total protein levels, while reduced infarcted volume of the brain and attenuated the brain tissues lipid peroxidation and calcium concentration in the brains of the rats in ischemia conditions induced by bilateral common carotid arteries occlusion. Additionally, the blood vessels congestions and neutrophil infiltration were observed which were accompanied with an increasing level of intercellular spaces due to ischemia in rats. All the damages were improved by the extract. The infarction area in the caudal side of the hippocampus was also reduced after treatment by the extract (Vekaria et al., 2012 ▶), (Table 4). 

***Linalool***

The possible neuroprotective effect of linalool was investigated in a glucose/serum deprivation-induced cytotoxicity model. Glucose/serum deprivation reduced cell viability when were cultured for 8 hr compared to the standard condition culture but treatment with 16 µg/ml of linalool improved cell viability (Alinejad et al., 2013 ▶). 

The effect of linalool on neuronal damages induced by acrylamide (ACR) was also evaluated. ACR is potentially a water-soluble neurotoxic monomer and is extensively used in different industries. In both human and animals, this monomer damages both the central nervous system (CNS) and peripheral nervous system (PNS). ACR-induced neurotoxicity has been suggested to be mainly due to oxidative stress. Administration of 12.5, 25, 50 and 100 mg/kg (i.p.) of linalool for 11 days significantly reduced severe gait abnormalities induced by ACR. Linalool also increased GSH content, however, it decreased lipid peroxidation in the cortical tissues of the rat brain (Mehri et al., 2015 ▶).

Linalool significantly reduced neuronal injury in the cortical tissues due to oxygen-glucose deprivation/reoxygenation (OGD/R), while it was not able to inhibit NMDA-induced excitotoxicity. It also significantly scavenged peroxyl radicals and reduced intracellular oxidative stress during OGD/R. Monocyte-chemoattractant protein-1 (MCP-1), as a chemokine which is released during OGD/R and induces microglial migration was also inhibited by linalool (Park et al., 2016a ▶), 

Linalool at lower concentrations (1, 2.5, and 5 µM) had no cytotoxic effects on SH-SY5Y cells, and did not significantly induce NO. Incubation of the cells with sodium nitroprusside (SNP, 2.5 mM) reduced cell viability. Linalool also increased neuronal cell viability (to 60% cell viability) and significantly prevented NO over-production. In addition, linalool significantly increased antioxidant levels and protected SH-SY5Y cells against SNP-induced cytotoxicity (Kim et al., 2015 ▶). It has been reported that linalool (10 μM) has therapeutic effects in treating ischemia-induced cerebral neuronal injury, by inhibition of oxidative stress and inflammatory responses in rat cortical cells (Park et al., 2016b ▶). Treatment of rats with linalool (12.5 mg/kg, i.p.) showed neuroprotective effects by reducing the progressive gait abnormalities promoted by ACR, oxidative stress, lipid peroxidation, and increasing the levels of GSH (Mehri et al., 2015 ▶), (Table 4).

***Beta-myrcene***

The effects of β-myrcene (a monoterpene derivated from coriander) on oxidative and histological damages induced by global cerebral ischemia/reperfusion (I/R) in tissues of C57BL/J6 mice brain, were investigated. Apoptosis in the tissues and other damages to the brain were revealed following I/R induction, while the antioxidant factors, including GSH, catalase, GPX and SOD were decreased. Treatment of animals by myrocin (MYR) (200 mg/kg, i.p.) for 10 days, significantly decreased thiobarbituric acid reactive substances (TBARS) formation. Furthermore, MYR protected against oxidative stress factors and significantly increased GSH, GPX, and SOD. Additionally, MYR showed a neuroprotective effect and decreased incidence of histopathological damages and apoptosis in the brain tissue (Ciftci et al., 2014 ▶), (Table 4).

**Table 4:** The effects of *C. sativum* and its components on neurotoxicity

**Extract/ Component**	**Type of study**	**Doses**	**Results**	**Ref.**
The leaves methanolic extract	Ischemic rat model	200 mg/kg, p.o.,	Increased total proteins, superoxide dismutase, catalase and glutathione while, attenuated cerebral infarcted volume, prevented form increasing of lipid peroxidation and calcium levels in the ischemic rat brain.	(Vekaria et al., 2012 ▶)
WF, EAF and NBF of *C. Sativum*	*In vitro*	0.1, 0.2, 0.4, and 0.8 mg/ml	WF at 0.4, 0.8 and 1.6 mg/ml significantly reduced glucose/serum deprivation-induced cytotoxicity.	(Ghorbani et al., 2011 ▶)
Linalool	*In vitro*	16 µg/ml	Significantly improved cell viability	(Alinejad et al., 2013 ▶)
Linalool	Acrylamide (ACR) induced neural damage in rat	12.5, 25, 50 and 100 mg/kg i.p.	Significantly reduced severe gait abnormalities induced by ACR	(Mehri et al., 2015 ▶)
Linalool	*In vitro*	0.01, 0.1, 1 and 10 µm	Significantly attenuated intracellular oxidative damages in OGD/R model. It also scavenged peroxyl radicals and also inhibited the monocyte-chemoattractant protein-1.	(Park et al., 2016a ▶)
Linalool	*In vitro*	1 µm, 2.5 µm, and 5 µm	Increased neuronal cell viability (about 60% cell viability) and significantly prevented from NO over- production. Linalool also improved antioxidant status.	(Kim et al., 2015 ▶)
Linalool	*In vitro*	10 μM	Showed therapeutically effects in treating ischemia-induced cerebral neuronal injury, by inhibition of oxidative stress and inflammatory responses.	(Park et al., 2016b ▶)
Linalool	ACR-induced neural damage in rat	12.5 mg/kg, i.p.	Showed neuroprotective effects by reducing the progressive gait abnormalities promoted by acrylamide, oxidative stress, lipid peroxidation, and increasing the levels of glutathione (GSH)	(Mehri et al., 2015 ▶)
Beta-myrcene (MYR)	Induced ischemia in mice	200 mg/kg; i.p.	Decreased in the antioxidant factors including, GSH, catalase, glutathione peroxidase (GPX) and SOD.	(Ciftci et al., 2014 ▶)

## Conclusion

Various parts of the plant, such as seeds and leaves, possess antioxidant, anxiolytic, antidepressive, learning and memory improving, neuroprotective, sedative-hypnotic, analgesic and anticonvulsant activities. Furthermore, linalool have different neuropharmacological effects, including anti-anxiety, sedative, anticonvulsant and anti-Alzheimer’s activities. Most of neuroprotective effects of *C. sativum* and its main component may be due to their antioxidant and anti-inflammatory properties. 
